# Availability of Higher-Level Neonatal Care in Rural and Urban US Hospitals, 2010-2022

**DOI:** 10.1001/jamanetworkopen.2025.59680

**Published:** 2026-02-12

**Authors:** Katy B. Kozhimannil, Emily C. Sheffield, Clara E. Busse, Julia D. Interrante, Corrie E. McDaniel, Sara C. Handley

**Affiliations:** 1 Division of Health Policy and Management, University of Minnesota School of Public Health, Minneapolis; 2 Department of Pediatrics, University of Washington, Seattle; 3 Seattle Children’s Hospital, Seattle, Washington; 4 Department of Pediatrics, University of Pennsylvania Perelman School of Medicine, Philadelphia; 5 Leonard Davis Institute of Health Economics, University of Pennsylvania, Philadelphia

## Abstract

This cohort study examines changes in the availability of higher-level neonatal care between 2010 and 2022 at rural and urban hospitals with childbirth services in the US.

## Introduction

Infant mortality is elevated for residents of rural US communities, in which access to childbirth care has been declining.^1,2^ Infants with high-acuity clinical needs have lower risks of mortality when treated in hospitals with higher-level neonatal care, which are often located in urban areas.^3,4,5^ We examined changes in availability of higher-level neonatal care at rural and urban hospitals with childbirth services between January 1, 2010, and December 31, 2022.

## Methods

This retrospective cohort study included all US hospitals that offered childbirth services (birth hospitals) and identified those with higher-level (intermediate or intensive) neonatal care. The study was deemed non–human participant research and exempt from review and informed consent by the University of Minnesota Institutional Review Board and followed the STROBE reporting guideline.

Using American Hospital Association annual surveys and Centers for Medicare & Medicaid Services Provider of Services files from 2010 to 2022, we applied an enhanced algorithm to identify birth hospitals.^6^ Birth hospitals were classified as having higher-level neonatal care if data indicated delivery of neonatal intensive care, at least 1 neonatal intensive care bed, or at least 1 neonatal intermediate care bed. Provider of Services files were used for confirmation. Primary reviews of hospital websites and news media were conducted to validate neonatal care status for hospitals with discrepancies between data sources, across years, and in cases of mergers and acquisitions.^6^

Hospital rurality was classified using Office of Management and Budget definitions. Urban hospitals were located in metropolitan statistical areas and rural hospitals in counties without an urbanized area of 50 000 inhabitants.

We assessed losses and gains of higher-level neonatal care from 2010 to 2022 by calculating the percentage of hospitals with higher-level neonatal care among birth hospitals open each year. We compared 2010 and 2022 percentages of hospitals with higher-level care using binomial generalized estimating equation models. Analyses were performed between May 20 and November 3, 2025, using SAS, version 9.4 (SAS Institute Inc) and Stata, version 18.0 (StataCorp LLC). *P* < .05 was considered significant.

## Results

There were 3257 US birth hospitals open at any point from 2010 to 2022 (1149 rural, 2108 urban). In 2010, 160 rural and 1281 urban birth hospitals offered higher-level neonatal care (Table). Between 2010 and 2022, 48 rural and 208 urban birth hospitals gained higher-level neonatal care, while 70 rural and 177 urban birth hospitals lost higher-level care. Rural areas saw a net loss of 22 birth hospitals offering higher-level neonatal care, and urban areas saw a net gain of 31 birth hospitals adding this care.

**Table.** Changes in Higher-Level (Intermediate or Intensive) Neonatal Care Availability at US Birth Hospitals, 2010-2022

Year	Short-term acute care hospitals, No.	Birth hospitals, No.					Birth hospitals with higher-level neonatal care, %^d^
		With obstetric and basic neonatal care^a^	Without higher-level neonatal care	With higher-level neonatal care	Gained higher-level neonatal care^b^	Lost higher-level neonatal care^c^	
**All hospitals**							
2010	4770	3126	1685	1441	NA	NA	46.1
2011	4770	3092	1655	1437	19	23	47.1
2012	4759	3061	1619	1442	38	33	48.4
2013	4753	3039	1588	1451	34	25	48.9
2014	4732	3005	1543	1462	32	21	49.7
2015	4718	2978	1511	1467	24	19	50.1
2016	4714	2944	1472	1472	24	19	50.8
2017	4708	2911	1432	1479	28	21	51.8
2018	4678	2866	1394	1472	19	26	52.0
2019	4657	2815	1340	1475	24	21	53.3
2020	4648	2758	1288	1470	20	25	54.0
2021	4654	2740	1263	1477	20	13	54.6
2022	4641	2699	1221	1478	15	14	55.3
Total^e^	4966	3257	NA	NA	256	247	NA
**Rural hospitals**							
2010	1937	1131	971	160	NA	NA	14.1
2011	1937	1106	958	148	2	14	13.6
2012	1936	1088	940	148	6	6	14.2
2013	1927	1077	924	153	9	4	15.0
2014	1916	1061	908	153	6	6	15.0
2015	1901	1043	892	151	6	8	15.1
2016	1897	1025	876	149	6	8	15.1
2017	1892	1009	862	147	6	8	15.2
2018	1874	982	840	142	4	9	14.9
2019	1864	955	810	145	6	3	15.8
2020	1856	929	780	149	5	1	16.6
2021	1855	921	771	150	4	3	16.7
2022	1849	897	748	149	3	4	16.9
Total^e^	1964	1149	NA	NA	48	70	NA
**Urban hospitals**							
2010	2833	1995	714	1281	NA	NA	64.2
2011	2833	1986	697	1289	17	9	65.8
2012	2823	1973	679	1294	32	27	67.2
2013	2826	1962	664	1298	25	21	67.4
2014	2816	1944	635	1309	26	15	68.7
2015	2817	1935	619	1316	18	11	68.9
2016	2817	1919	596	1323	18	11	69.9
2017	2816	1902	570	1332	22	13	71.2
2018	2804	1884	554	1330	15	17	71.4
2019	2793	1860	530	1330	18	18	72.5
2020	2792	1829	508	1321	15	24	73.0
2021	2799	1819	492	1327	16	10	73.8
2022	2792	1802	473	1329	12	10	74.4
Total^e^	3002	2108	NA	NA	208	177	NA

Abbreviation: NA, not applicable.

^a^ A small number of children’s hospitals (all urban) provided higher-level neonatal care during all years but gained (or gained and lost) obstetric care during 2011 to 2022. These hospitals were included in the total short-term acute care birth hospitals with obstetric and basic neonatal care for each year (8 in 2010, 7 in 2011, 6 in 2012-2015, 5 in 2016, 4 in 2017-2018, 2 in 2019, and 1 in 2020-2022).

^b^ Hospitals without higher-level neonatal care in the previous year but with higher-level neonatal care in the indicated year.

^c^ Hospitals with higher-level neonatal care in the previous year but without higher-level neonatal care in the indicated year (either because the hospital closed its higher-level neonatal care unit or the hospital itself closed).

^d^ Calculated as the number of hospitals that gained higher-level neonatal care plus the number of hospitals with higher-level neonatal care divided by the number of birth hospitals in that year.

^e^ Among facilities open and operating as a short-term acute care hospital in any year during 2010 to 2022, 3257 total, 1149 rural, and 2108 urban hospitals had obstetric and basic neonatal care at some point; 1212, 97, and 1115, respectively, had continuous higher-level neonatal care during 2010 to 2022; 10, 4, and 6, respectively, had higher-level neonatal care in 2010 to 2022 but had a period within those years when the unit was closed; and 3241, 1745, and 1496, respectively, never had higher-level neonatal care. For gains and losses of higher-level neonatal care, 256 total, 48 rural, and 208 urban hospitals gained a unit during 2011 to 2022; 219, 59, and 160, respectively, had a unit in 2010 but lost it during 2011 to 2022; and 28, 11, and 17, respectively, gained a unit after 2010 but lost it by 2022.

In 2010, 160 of 1131 rural birth hospitals (14.1%) had higher-level neonatal care and in 2022, 152 of 897 (16.9%) did, a nonsignificant difference. Among urban birth hospitals, 1281 of 1995 (64.2%) had higher-level neonatal care in 2010, increasing significantly to 1341 of 1802 (74.4%) in 2022 (*P* = .01) (Figure).

**Figure.**
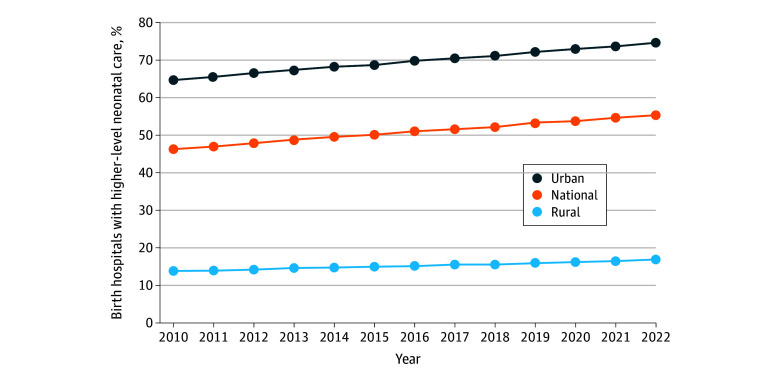
Percentage of US Birth Hospitals With Higher-Level (Intermediate or Intensive) Neonatal Care, 2010-2022 Percentages are based on the total number of birth hospitals each year (2010-2022). The numerator comprises all hospitals with higher-level neonatal care (including those that gained higher-level neonatal care) in a given year, and the denominator is the total number of hospitals with obstetric and basic neonatal care operating in that year. Denominators in 2010 were 3126 (national), 1131 (rural), and 1995 (urban). Denominators declined due to hospital closures and losses of obstetric and basic neonatal care, and in 2022, denominators were 2699 (national), 897 (rural), and 1802 (urban).

## Discussion

This cohort study suggests that access to higher-level neonatal care is limited at rural birth hospitals, as less than 20% offered this care in 2022 vs 74% of urban hospitals. While rural hospitals are losing childbirth care capacity,^2^ urban birth hospitals are expanding higher-level neonatal care, accentuating geographic discrepancies in access to care for high-risk infants.

Study limitations include that hospital data were self-reported and reported neonatal beds may not be used. The denominator for the study outcome decreased each year with hospital and obstetric unit closures, which were more prevalent among rural hospitals. While rurality is a continuum, we applied a dichotomous county-based measure. These hospital-level data do not contain patient-level information, precluding analysis of how higher-level neonatal care changes influenced patient outcomes.

Rural communities have less access to childbirth and higher-level neonatal care than urban communities. US infant mortality rates increase with the degree of rurality^1^; therefore, rural-urban differences in higher-level neonatal care availability may contribute to the survival gap for rural infants.

## Notes

Supplement.
Data Sharing Statement
