# Supplementary material for: Availability of Higher-Level Neonatal Care in Rural and Urban US Hospitals, 2010-2022
Source: JAMA Netw Open. 2026 Feb 12;9(2):e2559680. doi: 10.1001/jamanetworkopen.2025.59680 (PMC12902888; doi:10.1001/jamanetworkopen.2025.59680)
Supplement: Supplement. — Data Sharing Statement [file jamanetwopen-e2559680-s001.pdf]

Data Sharing Statement

Data
Data available: No

Additional Information
Explanation for why data not available: The data are only available through a data use agreement, and we are not able to share these data publicly.
